# Dual impacts of coronavirus anxiety on mental health in 35 societies

**DOI:** 10.1038/s41598-021-87771-1

**Published:** 2021-04-26

**Authors:** Sylvia Xiaohua Chen, Jacky C. K. Ng, Bryant P. H. Hui, Algae K. Y. Au, Wesley C. H. Wu, Ben C. P. Lam, Winnie W. S. Mak, James H. Liu

**Affiliations:** 1 The Hong Kong Polytechnic University, Hong Kong, China; 2 Hong Kong Shue Yan University, Hong Kong, China; 3 The University of New South Wales, Sydney, Australia; 4 The Chinese University of Hong Kong, Hong Kong, China; 5 Massey University, Auckland, New Zealand; 6 Department of Applied Social Sciences, Hong Kong Polytechnic University, Hung Hom, Kowloon, Hong Kong

**Keywords:** Psychology, Risk factors

## Abstract

The spread of coronavirus disease 2019 (COVID-19) has affected both physical health and mental well-being around the world. Stress-related reactions, if prolonged, may result in mental health problems. We examined the consequences of the COVID-19 pandemic on mental health in a multinational study and explored the effects of government responses to the outbreak. We sampled 18,171 community adults from 35 countries/societies, stratified by age, gender, and region of residence. Across the 35 societies, 26.6% of participants reported moderate to extremely severe depression symptoms, 28.2% moderate to extremely severe anxiety symptoms, and 18.3% moderate to extremely severe stress symptoms. Coronavirus anxiety comprises two factors, namely *Perceived Vulnerability* and *Threat Response*. After controlling for age, gender, and education level, perceived vulnerability predicted higher levels of negative emotional symptoms and psychological distress, whereas threat response predicted higher levels of self-rated health and subjective well-being. People in societies with more stringent control policies had more threat response and reported better subjective health. Coronavirus anxiety exerts detrimental effects on subjective health and well-being, but also has the adaptive function in mobilizing safety behaviors, providing support for an evolutionary perspective on psychological adaptation.

## Introduction

The COVID-19 outbreak in Wuhan, China was reported to the World Health Organization (WHO) on December 31, 2019, and on March 11, 2020, the WHO declared it a global pandemic. The speed of its worldwide transmission, the scope of the cross-industry impact, and the intensity of the media coverage have been unprecedented. Unlike influenza, COVID-19 is a new disease that has many unknowns. Due to the lack of confirmed antivirals, lack of vaccines during an early period of outbreaks, and unpredictable contagion, the COVID-19 pandemic has created worry and fear among the general public. The present research investigates the impacts of coronavirus anxiety on well-being indicators and takes an evolutionary approach to understanding the function of the anxiety response as well as its mechanisms at both the individual and society levels.

### Detrimental effects

Among the emotional responses to a pandemic, fear is a central reaction to the threat of or an actual occurrence of a pandemic^1^. Infectious diseases induce health-related fear, causing significant psychological unrest, as an infection is transmissible, imminent, and invisible^2^. People who are highly anxious about contracting COVID-19 may experience elevated levels of emotional distress and avoidance behaviors. Excessive fear or anxiety results in clinical conditions. Anxiety disorders such as generalized anxiety disorder and panic disorder are often accompanied by physical symptoms^3^. Previous work has investigated physiological arousal symptoms when exposed to information about coronavirus, such as dizziness, sleep disturbance, tonic immobility, appetite loss, and nausea or abdominal distress^4^.

During the initial stage of the COVID-19 outbreak in China, more than half of the respondents experienced moderate to severe negative emotional symptoms, and one-third reported moderate to severe anxiety^5^. The fear of COVID-19 was associated with depression, anxiety, and perceived vulnerability to disease in Iran^6^. People in Spain^7^ and Italy^8^ also experienced psychological distress following the outbreak. In the US, individuals with dysfunctional anxiety over COVID-19 exhibited impairment, alcohol/drug coping, negative religious coping, extreme hopelessness, and suicidal ideation^4^. These country-specific analyses have revealed the maladaptive psychological responses to the pandemic. Exposing public health crises, such as the Ebola outbreak and the severe acute respiratory syndrome (SARS), can cause mental health problems^9,10^. Persistent worries or concerns about COVID-19 occur with negative mental health outcomes. The prevalence of depression and anxiety is much higher in the presence of the pandemic^11^. Based on the clinical conceptualization of its detrimental effects and results of empirical studies from different regions, we hypothesize that coronavirus anxiety would negatively predict subjective well-being and positively predict negative emotional symptoms and psychological distress across cultures. In addition to a priori hypothesis, this research also explores age, gender, and cultural differences, as well as individual differences in coronavirus anxiety, and government responses to pandemic control.

### Adaptive function

The theory of evolution posits behaviors or traits that can improve survival and reproduction as functional products of natural selection^12^. Organisms develop the capacity to defend themselves against a wide variety of demands and threats in their environment. In the face of immediate danger, the emotion of fear mobilizes bodily resources to evoke a fight or flight response. Such mechanism enables prompt actions to cope with changing ecologies and enhance organisms’ survival probability. From an evolutionary perspective, the human anxiety response has an adaptive function. Anxiety arises from the perception of uncontrollable or unavoidable threats and emerges within the evolved context of defensive motivational systems^13^. It serves the functions of enhancing perceptual vigilance to detect potential threats, appraising routes and options to cope with flexibility, adjusting metabolic resources to prepare for action or avoidance, and consolidating learning through reinforcement and memory.

While excessive fear of infectious diseases may cause massive disruptions and impair functioning, fear appeals can activate defensive reactions and produce behavioral changes to deal with a health threat^14^. These responses can be adaptive if they involve focused attention, containment measures, and precautionary actions. Vigilance and civic responsibility are observed to be critical factors contributing to controlling the spread in East Asian societies, especially the practice of wearing face masks and compliance with social distancing regulations^15^. Social distancing measures and changes in population behavior are associated with reduced transmission in the community^16^. By and large, previous research on COVID-19 has documented its deleterious effects on mental well-being, but the psychological adaptation of coronavirus anxiety is not evident. We take an evolutionary approach to examining the adaptive function of coronavirus anxiety. In this research, we focus on the general population’s concerns about the spread of the coronavirus, the perceived likelihood of contracting the coronavirus, the avoidance of certain places and people, and the use of safety behaviors^17^. We conceptualize coronavirus anxiety as an emotional state characterized by worried thoughts and behavioral changes in response to the COVID-19 outbreak.

### Individual differences and government responses

To identify antecedents of pandemic-related anxiety, we will test the effect of a psychological variable that characterizes stable individual differences, such as the need for cognitive closure^18^, which refers to the desire to seek certainty and firm answers. Individuals who have a stronger need for cognitive closure desire secure and stable knowledge. The intolerance of uncertainty is likely to induce anxiety and fear^19^ and is associated with excessive worry, health anxiety, and mental disorders, including mood and anxiety disorders, and obsessive–compulsive disorder^3^. People with a strong need for closure have preference for predictability and discomfort with ambiguity, which would make them prone to coronavirus anxiety. In addition to individual differences, the present study also examines age, gender, and education level differences in coronavirus anxiety. We investigate the impacts of coronavirus anxiety on mental health indicators, including negative emotional symptoms (viz., depression, anxiety, and stress) and psychological distress across the globe. This research also examines its impact on self-rated health and subjective well-being (viz., self-esteem and life satisfaction).

As COVID-19 has spread, governments and health organizations have taken a wide range of measures to prevent possible transmission. Researchers use epidemic models to predict the outbreak and inform policy makers about the implementation of containment measures^20–22^. The outcomes of such measures entail empirical data to test their effectiveness. To evaluate the effects of government policies and public health interventions on citizens’ well-being, we conducted a multinational study to collect data from 35 countries/societies spanning all inhabited continents and diverse cultural zones. This design enabled us to test country-level predictors, such as COVID-19 severity including total numbers of confirmed cases, deaths, recoveries, and tests in each society, the Stringency Index^23^ capturing governments’ responses to the coronavirus through containment and closure policies, and the human development index (HDI) reflecting a society’s average achievement on social and economic dimensions.

## Results

### Data summary

We collected data from 25,065 community adults from 9–20 April, 2020, in 35 countries/societies from Asia, Europe, North America, South America, Oceania, and Africa. We partnered with an international data collection company Kantar, which curates a massive pool of potential participants around the world with over 88 million individuals. The panel method has the advantage of offering quick access to large and diverse samples, especially during the pandemic when a household survey is not feasible, and standardized data collection processes that make studies easy to replicate^24^. The final sample was generated from the pool based on stratified sampling techniques to create samples whose demographics (i.e., age, gender, and region within each country/society) closely matched those reported by the United Nations (UN) database: http://data.un.org/Host.aspx?Content=About.

To ensure data quality, we included three directed questions^25^ for attention checks (e.g., “This is a control question. Select ‘Agree’ and move on.”), which were distributed throughout the questionnaire. Participants who failed any of the three directed questions were removed from the survey, and thus the final sample consisted of 18,171 participants (50.2% female, *M*_*age*_ = 43.66, *SD* = 15.97, age range 18 to 91) with a completion rate of 71.6%. The average sample size for each country/society was 519, ranging from 507 in New Zealand to 530 in Brazil.

### Weighting

To better represent the underlying population, poststratification adjustment was employed to compensate for noncoverage issues through weighting. The weighted estimates are expected to be more similar to the underlying population; thus, the analyses in this research were performed on the weighted sample. We used a raking procedure to create the weighted sample^24,26^. Raking is an iterative proportion procedure, and it adjusts a sample distribution to match with a known population distribution. Using the Demographic Statistics Database extracted from the UN Statistics Division as the standard, analytical weight was created based on the marginal distributions of three demographic variables (age × gender × marital status) across the 35 societies. We paired each of the two gender groups (male and female) with each of the four age groups (18 to 29, 30 to 44, 45 to 59, and 60 + years) and each of the three marital status groups (single, married, and others), resulting in 24 categories to create the analytical weight for this study.

### Factor analysis and target rotation

We first examined the factor structure underlying the nine items of the Coronavirus Anxiety Inventory adapted from the Swine Flu Anxiety Inventory^17^, and performed exploratory factor analysis on the entire sample with oblique rotation. Parallel analysis suggested two factors, accounting for 64% of the total variance (see Table S1 in supplementary appendix). We regarded factor loadings greater than 0.30 as non-trivial factor loadings. As such, four items loaded on Factor 1, while three items loaded on Factor 2. Two items were dropped because they had double-loadings, retaining seven items in total. The items loading on the first factor reflected worried thoughts about coronavirus, and this was labeled *Perceived Vulnerability*. The items loading on the second factor reflected behavioral changes in response to coronavirus, and this was labeled *Threat Response*. To ensure that the results of the factor structure extracted from the entire sample were adequately represented in each society, Procrustes rotation was performed^27^. The factorial agreement of the two factors was generally supported across the 35 societies (see Table S2 in supplementary appendix).

### Severity of mental health problems

The descriptive statistics for the mental well-being indicators are reported in Table 1. Using the severity ratings of the 21-item Depression Anxiety and Stress Scale^28^, we computed the percentages of normal, mild, moderate, severe, and extremely severe levels of depression, anxiety, and stress symptoms for the entire weighted sample and for each society (see Tables S3 to S5 in supplementary appendix). Across the 35 societies, 12.3% of participants reported mild depression, 14.6% moderate depression, 6.0% severe depression, and 6.0% extremely severe depression; 7.6% reported mild anxiety, 14.0% moderate anxiety, 5.1% severe anxiety, and 9.1% extremely severe anxiety; 10.0% reported mild stress, 9.6% moderate stress, 6.2% severe stress, and 2.5% extremely severe stress.

**Table 1:** Descriptive statistics for mental well-being indicators across countries/societies.

	SRH		SE		LS		DEP		ANX		STR		PD	
	*M*	*SD*	*M*	*SD*	*M*	*SD*	*M*	*SD*	*M*	*SD*	*M*	*SD*	*M*	*SD*
Argentina	2.96	0.64	4.92	1.55	4.81	1.36	0.60	0.64	0.40	0.52	0.76	0.65	1.85	0.79
Australia	2.83	0.74	4.26	1.60	4.65	1.54	0.66	0.73	0.43	0.57	0.69	0.69	1.82	0.89
Brazil	3.01	0.66	4.90	1.64	4.62	1.53	0.82	0.73	0.57	0.63	1.03	0.77	2.17	0.92
Canada	2.85	0.72	4.48	1.49	4.69	1.51	0.72	0.73	0.49	0.58	0.79	0.69	1.92	0.89
China	2.78	0.61	5.33	1.03	4.62	1.32	0.66	0.69	0.63	0.65	0.82	0.64	1.87	0.77
Egypt	2.95	0.61	5.42	1.38	4.94	1.54	0.83	0.67	0.69	0.57	1.14	0.66	2.32	0.92
Finland	2.63	0.72	4.67	1.59	4.65	1.38	0.69	0.66	0.43	0.49	0.72	0.64	2.02	0.90
France	2.86	0.65	3.97	1.40	4.74	1.31	0.60	0.61	0.34	0.47	0.64	0.61	1.70	0.74
Germany	2.72	0.74	4.46	1.52	4.70	1.46	0.55	0.62	0.37	0.50	0.69	0.62	1.83	0.83
Hong Kong	2.51	0.59	4.55	1.16	3.99	1.39	0.69	0.63	0.53	0.50	0.82	0.58	1.84	0.76
India	3.12	0.64	5.45	1.46	5.26	1.31	0.88	0.78	0.84	0.74	0.99	0.75	2.25	0.99
Indonesia	2.97	0.63	5.74	1.23	5.01	1.43	0.45	0.58	0.54	0.52	0.80	0.61	2.08	0.84
Italy	2.75	0.66	4.61	1.39	4.07	1.38	0.65	0.62	0.34	0.49	0.69	0.64	2.07	0.83
Japan	2.48	0.75	4.07	1.34	3.80	1.52	0.54	0.59	0.29	0.39	0.61	0.59	1.67	0.72
Malaysia	2.96	0.61	5.23	1.40	4.98	1.34	0.65	0.67	0.56	0.55	0.78	0.62	1.91	0.75
Mexico	3.06	0.62	5.60	1.30	5.15	1.31	0.48	0.60	0.42	0.52	0.64	0.59	1.65	0.69
Netherlands	2.80	0.67	4.74	1.29	5.32	1.17	0.49	0.55	0.31	0.44	0.58	0.59	1.67	0.78
New Zealand	2.76	0.71	4.30	1.50	4.88	1.40	0.57	0.61	0.35	0.46	0.62	0.61	1.66	0.76
Nigeria	3.41	0.54	5.69	1.32	4.99	1.35	0.44	0.54	0.43	0.50	0.61	0.52	1.67	0.71
Pakistan	3.17	0.67	5.11	1.51	5.18	1.37	0.68	0.64	0.64	0.63	0.79	0.63	1.97	0.85
Philippines	3.03	0.62	5.20	1.35	5.11	1.32	0.65	0.62	0.65	0.58	0.79	0.56	1.93	0.81
Portugal	2.82	0.69	4.30	1.41	4.77	1.36	0.68	0.57	0.42	0.47	0.93	0.60	2.00	0.72
Russia	2.54	0.62	4.08	1.54	4.09	1.46	0.73	0.61	0.48	0.53	0.96	0.70	2.00	0.79
South Africa	3.12	0.69	4.97	1.61	4.52	1.47	0.70	0.69	0.51	0.57	0.82	0.64	1.98	0.88
South Korea	2.38	0.60	4.63	1.37	3.80	1.25	0.72	0.60	0.48	0.48	0.85	0.57	1.96	0.75
Singapore	2.74	0.57	4.52	1.36	4.63	1.38	0.66	0.67	0.51	0.52	0.76	0.61	1.87	0.85
Spain	2.89	0.68	4.62	1.51	4.55	1.48	0.49	0.59	0.33	0.42	0.63	0.60	1.93	0.82
Sweden	2.47	0.81	4.47	1.56	4.46	1.40	0.74	0.74	0.46	0.56	0.77	0.69	1.87	0.88
Taiwan	2.43	0.61	4.82	1.17	3.88	1.34	0.63	0.60	0.52	0.50	0.80	0.57	1.84	0.77
Thailand	2.74	0.63	5.75	1.01	4.98	1.27	0.66	0.60	0.67	0.54	0.95	0.60	2.21	0.80
Turkey	3.04	0.59	5.68	1.17	4.28	1.70	0.78	0.67	0.48	0.54	0.86	0.66	2.29	0.89
UAE	3.20	0.63	5.62	1.35	5.11	1.55	0.70	0.67	0.60	0.59	0.86	0.63	2.07	0.85
UK	2.79	0.70	3.93	1.65	4.77	1.47	0.66	0.69	0.41	0.52	0.71	0.63	1.81	0.84
USA	2.97	0.69	4.61	1.63	4.73	1.56	0.67	0.75	0.51	0.65	0.76	0.73	1.87	0.92
Vietnam	2.76	0.58	5.51	1.39	4.42	1.61	0.55	0.61	0.57	0.53	0.81	0.62	2.01	0.77

*SRH* self-rated health, *SE* self-esteem, *LS* life satisfaction, *DEP* depression, *ANX* anxiety, *STR* stress, *PD* psychological distress.

### Government responses and citizens’ well-being

To examine associations among well-being indicators, coronavirus anxiety, COVID-19 statistics, and the HDI (obtained from the United Nations Development Programme), and the Stringency Index (using the Oxford COVID-19 Government Responses Tracker). We performed correlation analyses at the society level.

**Figure 1:**
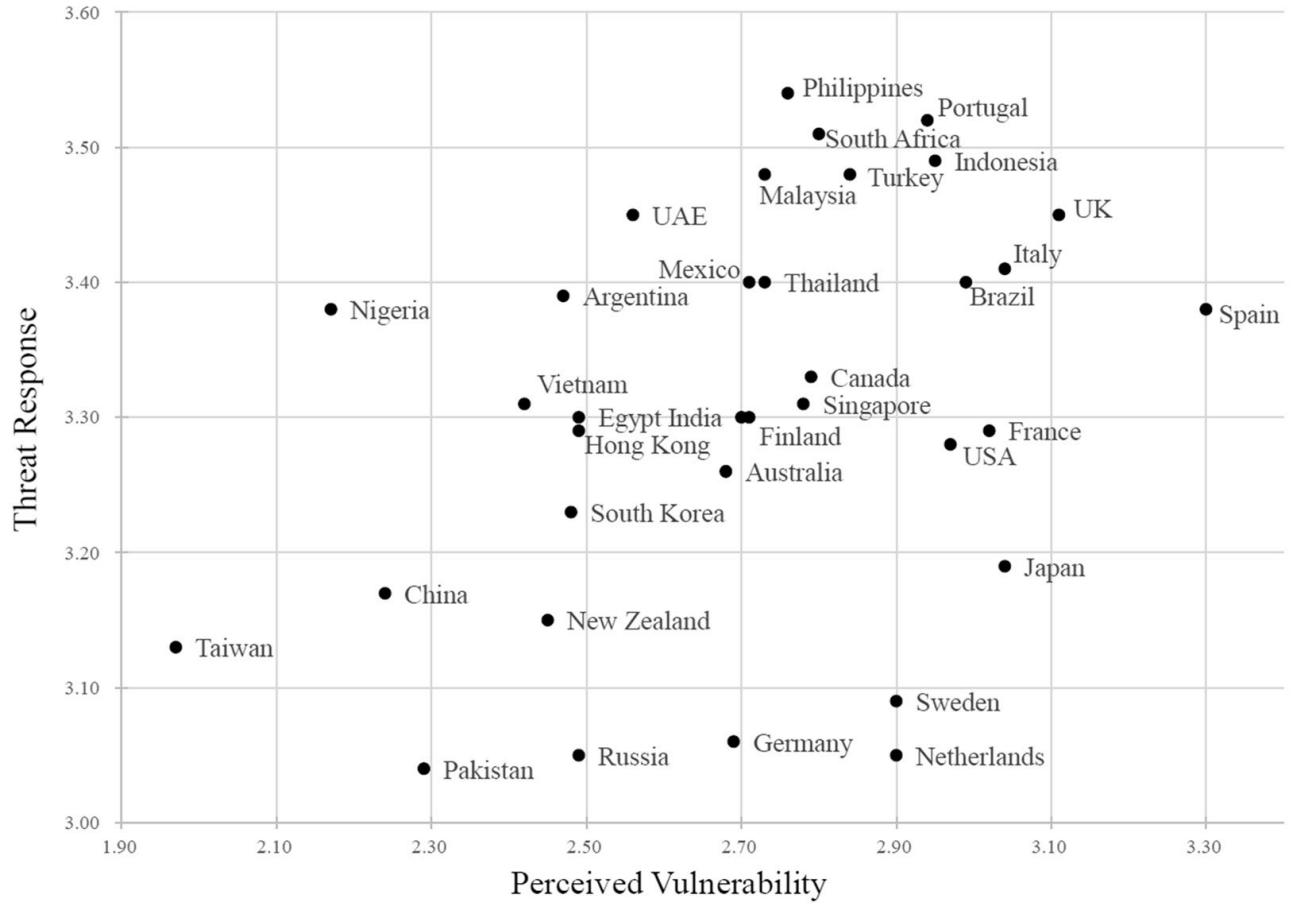
The country/society means of perceived vulnerability and threat response.

Figure 1 shows the relative position of each society by the intersection of perceived vulnerability and threat response. Citizens’ perceived vulnerability and threat response reflect their governments’ control strategies. Clustering regions of early interventions, such as Taiwan, China, and New Zealand, showed relatively low coronavirus anxiety on both factors, whereas clustering regions of less strict measures, such as Spain, UK, and Italy, showed relatively high coronavirus anxiety on both factors at the time of testing.

At the society level, we examined the correlations among COVID-19 statistics, the Stringency Index, the HDI, mental well-being indicators, perceived vulnerability and threat response (see Table 2). The total number of tests per thousand was negatively correlated with self-rated health, *r* = − 0.37, *p* = 0.039, and subjective well-being, *r* = − 0.52, *p* < 0.01. The Stringency Index was positively associated with self-rated health, *r* = 0.52, *p* = 0.001, and marginally with subjective well-being, *r* = 0.33, *p* = 0.054. Citizens in societies that performed more testing were less satisfied with their health and well-being, but those in societies with more stringent control policies reported better subjective health. Perceived vulnerability was positively correlated with the total number of confirmed cases per million, *r* = 0.61, *p* < 0.001, the total number of confirmed deaths per million, *r* = 0.58, *p* < 0.001, and the total number of recoveries per million, *r* = 0.36, *p* = 0.032, all of which represented the severity of infection with COVID-19 in a society. It was not related to the Stringency Index, but was positively related to HDI, *r* = 0.46, *p* < 0.01, indicating that citizens in developed countries/societies perceived more vulnerability than those in developing countries/societies. Threat response was not significantly correlated with the total number of confirmed cases, deaths, or recoveries, but was positively related to the Stringency Index, *r* = 0.34, *p* = 0.048, such that citizens in societies with tighter containment measures had more threat response.

**Table 2:** Correlations among coronavirus anxiety, mental well-being, and society-level indicators.

	Perceived vulnerability	Threat response	Self-rated health	Subjective well-being	Negative emotional symptoms	Psychological distress
Total number of confirmed cases (per million)	0.61***	0.02	− 0.10	− 0.31	− 0.43*	− 0.10
Total number of deaths (per million)	0.58***	0.06	− 0.07	− 0.27	− 0.43*	− 0.10
Total number of recoveries (per million)	0.36*	− 0.03	− 0.14	− 0.28	− 0.35*	− 0.05
Total number of tests (per thousand)	0.27	− 0.17	− 0.37*	− 0.52**	− 0.28	− 0.12
Stringency Index	0.21	0.34*	0.52**	0.33	0.08	0.25
Human Development Index	0.46**	− 0.22	− 0.67***	− 0.68***	− 0.37*	− 0.33

*p < 0.05, **p < 0.01, ***p < 0.001.

### Effects of psychological and demographic characteristics on coronavirus anxiety

Descriptive statistics and Cronbach’s alpha of perceived vulnerability and threat response are shown in the supplementary appendix (see Table S2). Participants in Spain scored highest (*M* = 3.30, *SD* = 0.50) in perceived vulnerability and those in Taiwan scored the lowest (*M* = 1.97, *SD* = 0.66), whereas participants in the Philippines scored highest (*M* = 3.54, *SD* = 0.61) in threat response and those in Pakistan scored the lowest (*M* = 3.04, *SD* = 0.74).

Since the present data had a two-level structure with individuals nested within countries/societies, the issue of data dependency within societies was addressed by performing multilevel structural equation modeling, such that variables were broken down into within- and between-level variations^29,30^. This set of analysis focused on how individuals’ levels of need for cognitive closure as well as demographic variables (age, gender, and education level) were associated with coronavirus anxiety, and hence multilevel regression was conducted to examine effects at the within-country level (individual level) after accounting for between-country variation. Results at the between-country level (country/society level) are reported in the supplementary appendix (see Table S6). Models were tested with random intercepts and fixed slopes across the 35 societies.

Multilevel regression was conducted to examine the prediction of need for cognitive closure (NFC) on perceived vulnerability and threat response (see Table 3), controlling for the covariates of age, gender, and education level. First, age did not predict perceived vulnerability, *b* = − 0.00, β = − 0.02, *p* = 0.160, but positively predicted threat response, *b* = 0.00, β = 0.04, *p* = 0.001, indicating that old adults reported higher levels of threat response than younger adults. Gender (male = 1, female = 2) positively predicted both perceived vulnerability, *b* = 0.09, β = 0.07, *p* < 0.001, and threat response, *b* = 0.12, β = 0.09, *p* < 0.001. Compared with male participants (*M* = 2.65, *SD* = 0.71 for perceived vulnerability; *M* = 3.25, *SD* = 0.73 for threat response), females scored higher in both factors of coronavirus anxiety (*M* = 2.76, *SD* = 0.68 for perceived vulnerability; *M* = 3.36, *SD* = 0.68 for threat response). Education level (below high school or others = 1; high school = 2; above high school = 3) positively predicted both perceived vulnerability, *b* = 0.05, β = 0.04, *p* < 0.001, and threat response, *b* = 0.11, β = 0.08, *p* < 0.001, indicating that participants with higher levels of education scored higher in both factors of coronavirus anxiety than those with lower levels of education.

**Table 3:** Multilevel regression predicting perceived vulnerability and threat response by need for cognitive closure at the individual level.

	Perceived vulnerability	Threat response
	β	β
Age	− 0.02	0.04**
Gender ^a^	0.07***	0.09***
Education level	0.04***	0.08***
Need for cognitive closure	0.10***	0.10***

**p < 0.01, ***p < 0.001.

^a^Male = reference group.

NFC positively predicted both perceived vulnerability, *b* = 0.10, β = 0.10, *p* < 0.001, and threat response, *b* = 0.10, β = 0.10, *p* < 0.001. This supports our prediction that stronger need for cognitive closure is linked to higher coronavirus anxiety. Additional models with the random effects of NFC on perceived vulnerability and threat response were tested, and their estimates were consistent with those of fixed effects. The random slope from NFC to perceived vulnerability was not significant, *b* = 0.001, *p* = 0.106, while the random slope from NFC to threat response was significant but negligible, *b* = 0.000, *p* = 0.048. Thus, the effects of NFC on coronavirus anxiety were generally invariant across societies.

### Effects of coronavirus anxiety on well-being

To examine the associations of coronavirus anxiety with mental well-being indicators at the individual level, multilevel correlations analyses were first performed, followed by multilevel regression analyses. Results on the between-country level effects are reported in the supplementary appendix (see Table S7). The results of multilevel correlations are presented in Table 4. First, perceived vulnerability was positively correlated with threat response, *r* = 0.42, *p* < 0.001. Second, perceived vulnerability was negatively correlated with the positive indicators of one’s well-being (e.g., self-rated health and life satisfaction) while being positively correlated with the negative indicators of one’s well-being (e.g., anxiety and psychological distress). Third, threat response was positively correlated with most of the indicators of one’s well-being.

**Table 4:** Multilevel correlations of perceived vulnerability and threat response with well-being outcomes.

	1	2	3	4	5	6	7	8	9
1. Perceived vulnerability	–	0.36*	0.01	− 0.34*	0.05	− 0.04	− 0.38*	− 0.19	0.07
2. Threat response	0.42***	–	0.50**	0.38*	0.21	0.04	0.17	0.18	0.35*
3. Self-rated health	− 0.12***	0.01	–	0.58***	0.72***	− 0.05	0.32*	0.06	0.19
4. Self-esteem	− 0.00	0.09***	0.26***	–	0.43**	− 0.02	0.63***	0.34	0.48*
5. Life satisfaction	− 0.07***	0.05***	0.43***	0.31***	–	− 0.12	0.33	0.00	0.04
6. Depression	0.17***	− 0.03**	− 0.29***	− 0.26***	− 0.42***	–	0.57*	0.77**	0.66**
7. Anxiety	0.19***	0.01	− 0.25***	− 0.14***	− 0.25***	0.73***	–	0.75***	0.65**
8. Stress	0.19***	0.05***	− 0.24***	− 0.18***	− 0.32***	0.78***	0.77***	–	0.83***
9. Psychological distress	0.19***	0.02*	− 0.29***	− 0.24***	− 0.40***	0.78***	0.68***	0.74***	–

Correlations at the individual level are provided below the diagonal while those at the society level are provided above the diagonal.

*p < 0.05, **p < 0.01, ***p < 0.001.

Given that perceived vulnerability and threat response were moderately associated, *r* = 0.42, *p* < 0.001, multilevel regression models were established to examine the joint predictions from the two factors on individuals’ mental well-being (see Table 5). In addition to the covariation between perceived vulnerability and threat response, we included age, gender, and education level as covariates. Results showed that age negatively predicted self-rated health, *b* = − 0.01, β = − 0.11, *p* < 0.001, negative emotional symptoms, *b* = − 0.01, β = − 0.20, *p* < 0.001 and psychological distress, *b* = − 0.01, β = − 0.22, *p* < 0.001, and positively predicted subjective well-being, *b* = 0.01, β = 0.11, *p* = 0.009. These results revealed that older adults evaluated their health conditions less satisfactorily, but reported better well-being and less emotional distress than younger adults. Gender negatively predicted self-rated health, *b* = − 0.04, β = − 0.03, *p* = 0.009 and subjective well-being, *b* = − 0.06, β = − 0.03, *p* = 0.012, while positively predicting negative emotional symptoms, *b* = 0.04, β = 0.04, *p* = 0.002 and psychological distress, *b* = 0.11, β = 0.07, *p* < 0.001, indicating that compared with male participants, females experienced lower levels of subjective health and well-being, and higher levels of emotional distress. Education level positively predicted self-rated health, *b* = 0.08, β = 0.07, *p* < 0.001, and subjective well-being, *b* = 0.18, β = 0.08, *p* < 0.001, while negatively predicting negative emotional symptoms, *b* = − 0.05, β = − 0.05, *p* < 0.001, and psychological distress, *b* = − 0.07, β = − 0.05, *p* < 0.001. Participants with higher levels of education reported higher levels of subjective health and well-being, and lower levels of emotional distress than those with lower levels of education.

**Table 5:** Multilevel regression predicting well-being outcomes by perceived vulnerability and threat response at the individual level.

	Self-rated health	Subjective well-being	Negative emotional symptoms	Psychological distress
	β	β	β	β
Age	− 0.11***	0.11***	− 0.20***	− 0.22***
Gender ^a^	− 0.03**	− 0.03*	0.04**	0.07***
Education level	0.07***	0.08***	− 0.05***	− 0.05***
Perceived vulnerability	− 0.16***	− 0.09***	0.23***	0.21***
Threat response	0.08***	0.12***	− 0.08***	− 0.06***

*p < 0.05, **p < 0.01, ***p < 0.001.

^a^Male = reference group.

Perceived vulnerability predicted lower levels of self-rated health, *b* = − 0.16, β = − 0.16, *p* < 0.001, and subjective well-being, *b* = − 0.16, β = − 0.09, *p* < 0.001, and higher levels of negative emotional symptoms, *b* = 0.20, β = 0.23, *p* < 0.001, and psychological distress, *b* = 0.27, β = 0.21, *p* < 0.001. Interestingly, threat response predicted higher levels of self-rated health, *b* = 0.07, β = 0.08, *p* < 0.001, and subjective well-being, *b* = 0.12, β = 0.08, *p* < 0.001, and lower levels of negative emotional symptoms, *b* = − 0.06, β = − 0.08, *p* < 0.001, and psychological distress, *b* = − 0.07, β = − 0.06, *p* < 0.001.

Additional models with the random effects of perceived vulnerability and threat response on mental well-being across societies were tested. Overall, all the estimates of random effects across societies were consistent with the estimates of fixed effects. None of the random slopes were significant except that there was a significant but negligible random slope from threat response to psychological distress, *b* = 0.003, *p* = 0.039. Thus, the effects of perceived vulnerability and threat response on mental well-being were generally invariant across societies. We also tested the interaction effects between perceived vulnerability and threat response on mental well-being and summarized the results in the supplementary appendix.

## Discussion

The present research revealed the prevalence of mental health problems in a sample stratified by age, gender, and region of residence across 35 societies during the COVID-19 pandemic. Overall, 26.6% of participants experienced moderate to extremely severe depression symptoms, 28.2% moderate to extremely severe anxiety symptoms, and 18.3% moderate to extremely severe stress symptoms, indicating the need for action on mental health. Coronavirus anxiety consists of two factors, namely perceived vulnerability and threat response. Perceived vulnerability delineates worries about the contagion of COVID-19 and the likelihood of being infected, whereas threat response captures behavioral responses to these fears, such as social avoidance and preventive practices. The moderate positive correlation between the two factors showed that both worried thoughts and behavioral changes reflect signs of anxiety related to COVID-19, but that they predicted outcome variables differently. Perceived vulnerability predicted lower levels of self-rated health and subjective well-being, and higher levels of negative emotional symptoms and psychological distress. In contrast, threat response predicted higher levels of self-rated health and subjective well-being, and lower levels of negative emotional symptoms and psychological distress.

In addition, we found age, gender, education, and individual differences in coronavirus anxiety. Women and individuals with higher levels of education reported higher levels of perceived vulnerability and threat response. This is supported in previous literature, which showed that education was positively correlated with coronavirus anxiety^4^. Older people also scored higher in threat response. The psychological factor of need for cognitive closure positively predicted both factors, such that individuals who have a general tendency of preferring order, predictability, and decisiveness are prone to coronavirus anxiety. People in developed countries perceived more vulnerability than those in developing countries, whereas those in societies with tighter containment measures had more threat response. People in societies that performed more testing were less satisfied with their health and well-being, but those in societies with more stringent control policies reported better subjective health.

Consistent with the results from different regions^5,11^, the present study has found higher prevalence rates of depression, anxiety, and stress during the COVID-19 pandemic. Across 35 societies, the findings support our a priori hypothesis, indicating that perceived vulnerability negatively predicted subjective well-being and positively predicted negative emotional symptoms and psychological distress across cultures. These results are also aligned with those obtained from single countries; for example, fear of COVID-19 was positively correlated with depression and anxiety in Iran^6^. Coronavirus anxiety was also significantly related to functional impairment and maladaptive coping in the US^4^. Though national surveys reported higher-than-usual levels of psychological distress during the pandemic, 76% – 85% of people with mental illness in low- and middle- income countries receive no treatment for their conditions around the world^31^. Evidence-based research is needed to mitigate the mental health consequences of the pandemic in the global context.

The dual impacts of coronavirus anxiety have important implications for clinical practice and intervention policy. Early research on COVID-19 has focused on its diagnosis, transmission, and treatment, as well as infection prevention and control. When the psychosocial consequences of the outbreak started to receive attention, the majority of empirical studies on mental health in single countries found detrimental effects^4,6^, and this is consistent with the results of perceived vulnerability found in our study. Anxiety is a normal emotion in the face of potentially harmful stressors such as the pandemic, leading to nervousness and apprehension^1^. Our results on threat response reveal the adaptive function of coronavirus anxiety and attest to its evolutionary aspects, that is, motivating appropriate precautionary actions to confront imminent threats. The ecological hazards brought by pathogenic diseases present selection pressures and profound challenges throughout human history, because of their fast transmission and high mortality^2^. Pathogen prevalence affects psychological phenomena and social behavior^3,32^. Anxious states activate defensive motivational systems to organize bodily resources and generate coping mechanisms to promote survival^13^. These behavioral changes are related to better subjective health, greater mental well-being, and less emotional distress, as shown in our study, demonstrating the psychological benefits of evolved adaptations. In this sense, our findings provided support for an evolutionary perspective on coronavirus anxiety. When excessive anxiety impairs daily functioning, however, the symptoms of anxiety disorders require psychiatric treatment.

Public health interventions may instigate a certain amount of illness-related anxiety to mobilize behavioral changes in the community. Threat appeals activate fear arousal in public health campaigns, but the greatest behavior change comes with high levels of efficacy^14^. Thus, health communications may improve effectiveness by using threat-based messages that present aversive outcomes of COVID-19 to elicit feelings of fear and appraisals of susceptibility, but offer specific, easily implementable recommendations to elicit threat response and perceived efficacy. There are various uncertainties about COVID-19, from unpredictable transmission to optimal treatment, people with a strong need for cognitive closure are difficult to tolerate uncertainty and thus experience distress^3,19^. Clear and consistent messages on infection prevention and control can also help these individuals to reduce ambiguity and anxiety, so that they can concentrate their attention on adaptive coping.

Our findings suggest that government responses to the COVID-19 outbreak were associated with citizens’ behavioral changes, be it voluntarily or involuntarily. At the society level, containment and closure policies measured by the Stringency Index were positively related to citizens’ use of safety behavior and social distancing. Containment and closure policies enforce school closures and restrictions in movement, to some extent forcing citizens to practice precautionary behaviors. At the individual level, these protective responses had perceived benefits to physical and mental health. The COVID-19 outbreak has brought burgeoning studies on modeling and forecasting the spread of the pandemic, which inform governments to make decisions on containment and closure policies. Yet, the effectiveness of these measures depends on the compliance of the general public. Negative emotions and response fatigue may backfire on control strategies^33^. Therefore, global health interventions based on mathematical modeling should factor in psychological reactions and mental health outcomes of citizens in different countries. A review shows that the negative psychological effects of quarantine are manifested in post-traumatic stress symptoms, confusion, and anger^33^. In addition to providing strong rationale, clear protocols, and sufficient supplies, promoting altruism and improving communication may mitigate the averse psychological impact of containment measures.

On the other hand, the HDI reflects economic affluence and is supposed to represent the capacity of a country’s healthcare system to cope with infectious diseases. Developed countries generally have greater medical resources than developing countries, such as medical staff, hospital beds, and intensive care facilities. However, a higher rank in the HDI was associated with higher perceived vulnerability, perhaps because the spread of contagion was serious in developed countries at the time of data collection. During the early period of the outbreak, faster spread of COVID-19 was found in countries with higher relational mobility^34^, which is characteristic of North America and Europe^35^. Regions with lower HDI are also predominated by collectivistic values that serve an antipathogen defense function, as their history of pathogen prevalence alerts them to maintain ritualized buffers against disease^32^. The total numbers of confirmed cases and deaths denote the severity of infection within a society, also related to perceived vulnerability. Further, the total number of tests reflects governments’ efforts to monitor the spread of virus, but is associated with citizens’ negative evaluation of physical and psychological health. It is possible that massive testing boosts the number of confirmed cases and accordingly amplifies media coverage.

This study has some limitations. At the time of data collection, countries had gone through different stages of outbreak and transmission, during which their citizens’ emotional responses fluctuated with the course of COVID-19 containment. Our data on mental well-being indicators could only reflect participants’ psychological states at a single time point. Though we analyzed the Stringency Index at the same time of data collection, containment and closure policies change over time and vary across societies, time-series data are needed to evaluate the effects of government measures on controlling the spread of the virus^36^. Moreover, as in all multinational research based on self-reports, the results could have been affected by response styles. Our findings remained robust after the index of extreme response style^37^ was computed and controlled for (see Tables S8 and S9 in the supplementary appendix), but cross-cultural comparisons have to be interpreted with caution. Another limitation is the representativeness of data, which is subject to the participants included in the study. The use of the panel method to collect survey data has been increasing in recent years, and multinational studies using Internet panels have been recognized in the field^38,39^. It is similar to many empirical studies recruiting undergraduates, such as in multicultural studies, and samples of patients receiving care at select sites of care^24^. Nevertheless, to obtain an unbiased representation of the total population, random sampling would be preferred so that each member has an equal probability of being chosen.

It is important to understand the extent of the mental health consequences of the pandemic, so as to strengthen advocacy efforts for mental health and ensure psychosocial support for recovery. We found that the effects of coronavirus anxiety on mental well-being were generally invariant across societies. Women and older adults are vulnerable groups that deserve specific attention. Practical help, resources, and services should be provided to assist these groups in overcoming their difficulties due to quarantine and lockdown. Considering cultural contexts and integrating mental health services with primary care may be a possible way to provide holistic interventions^40,41^. Our research shows that both individuals’ threat response and governments’ policy response are positively associated with mitigating the pandemic-related impact on mental well-being. Therefore, proactive responses at both the individual and society levels are important to the development of treatment plans, the implementation of psychosocial interventions, and support for community-based services.

## Methods

### Procedure

To examine the effects of our hypothesized exogenous variables on a given endogenous variable at the individual level, we conducted a power analysis. This indicated a per country/society sample size of 471 participants, yielding at least 80% of statistical power. The estimation assumed a small-to-moderate size of correlations ($\rho$ = 0.20) among exogenous variables and a small size of correlations ($\rho$ = 0.10) between exogenous variable and endogenous variable^42^.

Following the standard procedures of translation and back-translation in cross-cultural research^43^, translation from English to each of the 22 non-English languages was conducted by bilinguals of English and each non-English language, and then separate bilinguals conducted back-translation to English for each language version. We compared back-translation of each language version with the original English version and discussed the discrepancies, which ensured the accuracy or revision of the translation. Then the bilinguals in each country revised the translation to solve the discrepancies. We did pilot testing to examine the clarity of the questions and content.

To assess the prevalence of mental health problems across the globe, we administered well-validated measures, namely self-rated health^44^, subjective well-being (consisting of two indicators, self-esteem^45^ and life satisfaction adopted from the World Value Survey^46^), negative emotional symptoms consisting of three indicators assessed by the Depression Anxiety and Stress Scale (DASS-21)^28^ and psychological distress (K6)^47^, and need for cognitive closure^48^. To measure coronavirus-related anxiety, we adapted the Swine Flu Anxiety Inventory (SFI)^17^ which was based on clinical observations of clinicians and researchers evaluating and treating individuals with anxiety disorders and somatization. The SFI demonstrated sound psychometric properties and satisfactory internal consistency. It was positively associated with health-related anxiety, concerns about the likelihood and severity of contamination, disgust sensitivity, and the tendency to carefully monitor one’s internal bodily sensations, supporting the construct validity of Swine Flu anxiety.

Participants also reported demographic information, such as age, gender, and education level. Informed consent was obtained from all participants at the beginning of the study by indicating their willingness to participate in the study on the consent statement. Ethics approval for this study was obtained from the Human Subjects Ethics Sub-committee of the Department of Applied Social Sciences, Hong Kong Polytechnic University (#HSEARS20200402995). All methods were carried out in accordance with relevant guidelines and regulations. This study was pre-registered prior to data collection at the Open Science Framework: https://osf.io/9x3re/?view_only=94ac9c3f5a8f41c0b25e49b45826dfae.

### Measures

#### Depression, anxiety, and stress

The 21-item Depression Anxiety and Stress Scale (DASS-21)^28^ was employed to assess emotional distress in the previous week via three indicators: depression, anxiety, and stress. Responses were anchored on 4-point scales ranging from 0 (*did not apply to me at all*) to 3 (*applied to me very much, or most of the time*). Sample items included, “I felt that life was meaningless” (depression), “I felt scared without any good reason” (anxiety), and “I felt that I was rather touchy” (stress). Research on the DASS has documented high internal consistency and discriminant validity in a variety of settings, meeting the requirements for research and clinical use^28^. In this study, averaged Cronbach’s alphas = 0.88, 0.82, and 0.86, respectively. The cut-offs for normal, mild, moderate, severe, and extremely severe levels of depression, anxiety, and stress symptoms were obtained from the *Manual for the Depression Anxiety Stress Scales (2nd ed.)*^28^. The total score produced a composite measure of negative emotional symptoms^28^.

#### Self-rated health

The 3-item Self-Rated Health Status Measure^44^ was adapted to assess participants’ evaluation of their overall health on 4-point scales, ranging from 1 (*poor*) to 4 (*excellent*). A sample item is “How would you rate your overall health at the present time?” The measure achieved satisfactory reliability and validity as an indicator of global self-rated health in previous research^44^. Higher scores indicated better self-rated health. Averaged α = 0.85.

#### Self-esteem

The Single-Item Self-Esteem Scale^45^ was used to measure overall evaluation of self-worth (i.e., “I have high self-esteem”) on a 7-point scale, ranging from 1 (*not very true of me*) to 7 (*very true of me*). This shortened scale has proven to be as satisfactory in convergent and predictive validity as the 10-item Rosenberg Self-Esteem Scale^49^.

#### Life satisfaction

Adopted from the World Values Survey^46^, a single item “All things considered, how satisfied are you with your life as a whole these days?” was used to measure the evaluation of one’s own life with well-documented validity. Responses were anchored on a 7-point scale, ranging from 1 (*completely dissatisfied*) to 7 (*completely satisfied*).

#### Psychological distress

The 6-item Kessler Psychological Distress Scale (K6)^47^ was used to measure painful mental and physical symptoms that are associated with stressors in life. A sample item is “During the last 30 days, about how often did you feel restless or fidgety?” The items were rated on 5-point scales, ranging from 1 (*none of the time*) to 5 (*all of the time*). As K6 has shown good psychometric properties and ability to discriminate the Diagnostic and Statistical Manual of Mental Disorders, fourth edition (DSM-IV) cases from non-cases, it has been used in general-purpose health surveys^47^. Higher scores indicated greater psychological distress. Averaged α = 0.90.

#### Coronavirus anxiety

We adapted the 9-item Swine Flu Anxiety Inventory (SFI)^17^ to measure coronavirus anxiety, replacing “Swine Flu” with “coronavirus” and “U.S.” with “your country/society.” Participants rated their agreement with each item on 4-point Likert scales, ranging from 1 (*very little*) to 4 (*very much*). Two factors were extracted, namely perceived vulnerability (e.g., “How likely is it that you could become infected with coronavirus?”) and threat response (e.g., “To what extent has the threat of coronavirus influenced your use of safety behaviors [e.g., hand sanitizer]?”). The averaged Cronbach’s alphas for the two factors were 0.78 and 0.68, respectively, across the 35 societies.

#### Need for cognitive closure

The 15-item Need for Closure (NFC) Scale^48^ was used to measure the desire for closure and predictability rather than confusion and ambiguity. Items were rated on 6-point Likert scales, ranging from 1 (*strongly disagree*) to 6 (*strongly agree*). A sample item is “I don’t like situations that are uncertain.” This short version demonstrated similar psychometric properties and validity as the 41-item version revised by Roets and Van Hiel^50^, which was based on the 42-item NFC scale developed by Webster and Kruglanski^51^. Higher scores indicated stronger need for cognitive closure. Averaged α = 0.84.

### Data availability

Data on COVID-19 severity of infection were obtained from European Centre for Disease Prevention and Control (ECDC). Data on recoveries and in Hong Kong were obtained from the John Hopkins University CSSE database: https://github.com/CSSEGISandData. Data on total tests were obtained from https://ourworldindata.org/coronavirus-testing. Data on policy scores were obtained from the Oxford COVID-19 Government Responses Tracker (OxCGRT): https://www.bsg.ox.ac.uk/research/research-projects/coronavirus-government-response-tracker. The OxCGRT computed a Stringency Index based on eight indicators of containment and closure policies (e.g., school closures and restrictions in movement). Data on the Human Development Index (HDI) were obtained from the United Nations Development Programme.

As the United Nations does not have data for Taiwan, we extracted Taiwan’s demographic data from the Department of Household Registration Affairs, Ministry of the Interior, Taiwan to create analytical weight.

The HDI is the geometric mean of normalized indices for three dimensions with indicators, including health (life expectancy at birth), education (years of schooling), and standard of living (gross national income per capita). The Stringency Index comprises eight indicators, including school closures, workplace closures, the cancellation of public events, restrictions on size of gathering, closing public transport, stay-at-home requirements, restrictions on internal movement, and restrictions on international travel. Publicly available information and data on these indicators are collected and aggregated into a composite of indices to record the strictness of government policies in response to the COVID-19 outbreak.

## Supplementary Information

Supplementary Information.
