# Supplementary material for: Dual impacts of coronavirus anxiety on mental health in 35 societies
Source: Sci Rep. 2021 Apr 26;11:8925. doi: 10.1038/s41598-021-87771-1 (PMC8076265; doi:10.1038/s41598-021-87771-1)
Supplement: Supplementary file 1 — Supplementary Information. [file 41598_2021_87771_MOESM1_ESM.pdf]

Dual Impacts of Coronavirus Anxiety on Mental Health in 35 Societies

Sylvia Xiaohua Chen[1*], Jacky C. K. Ng[2], Bryant P. H. Hui[1], Algae K.Y. Au[1], Wesley C. H. Wu[1], Ben C. P. Lam[3], Winnie W. S. Mak[4], James H. Liu[5]

Affiliations:

[1]The Hong Kong Polytechnic University

[2]Hong Kong Shue Yan University

[3]The University of New South Wales

[4]The Chinese University of Hong Kong

[5]Massey University

Correspondence to: Sylvia Chen, Department of Applied Social Sciences, Hong Kong Polytechnic University, Hung Hom, Kowloon, Hong Kong; e-mail: ssxhchen@polyu.edu.hk.

Supplementary Appendix

Procrustes rotation

We performed Procrustes rotation[1] to examine whether the results of the factor structure extracted from the entire sample were adequately represented in each society. Specifically, the factor structure found in each society was target-rotated to the factor structure based on the entire sample. To evaluate the extent of factorial agreement, the congruence coefficient of Tucker's phi was computed[2]. A value of Tucker's phi greater than .90 suggests good factorial agreement. Results indicated that all societies yielded Tucker's phi greater than .90 for both factors, except for the Tucker's phi of threat response in Germany, which was smaller than .90. The average of Tucker's phi across societies was .99 for perceived vulnerability and .98 for threat response. Thus, the factorial agreement of the two factors was generally supported across the 35 societies.

Interaction between perceived vulnerability and threat response

We also tested additional models to explore the interaction effect between perceived vulnerability and threat response on mental well-being. Individuals' perceived vulnerability and threat response interacted to predict self-rated health, $b = 0.03$, $p = .011$, and subjective well-being, $b = 0.07$, $p < .001$, while the interaction effects were not significant for negative emotional symptoms, $b = 0.02$, $p = .115$, and psychological distress, $b = 0.02$, $p = .285$. Simple slope analysis indicated that individuals' threat response could weaken the negative effect of perceived vulnerability on self-rated health and subjective well-being. Among those who scored high in threat response (1 *SD* above mean value), the negative effects of perceived vulnerability on self-rated health, $b = -0.24$, $p < .001$, and subjective well-being, $b = -0.34$, $p < .001$, were weaker than the effects among those who scored low in threat response (1 *SD* below mean value), $b = -0.28$, $p < .001$ for self-rated health, and $b = -0.43$, $p < .001$ for subjective well-being.

References

1 van de Vijver FJR, Leung K. Methods and Data Analysis for Cross-Cultural Research. Thousand Oaks, CA: Sage, 1997.

2 Tucker LR. A Method for Synthesis of Factor Analysis Studies. Princeton NJ: Educational Testing Service, 1951.

Table S1. *Factor Loadings of the Items for the Coronavirus Anxiety Inventory*

Items	F1	F2
1. How likely is it that someone you know could become infected with coronavirus	**.87**	-.08
2. How likely is it that you could become infected with coronavirus	**.86**	-.09
3. How quickly do you believe contamination from coronavirus is spreading in your country/society	**.69**	.14
4. To what extent do you believe that coronavirus could become a “pandemic” in your country/society	**.63**	.27
5. If you did become infected with coronavirus, to what extent are you concerned that you will be severely ill [a]	**.42**	**.39**
6. To what extent are you concerned about coronavirus [a]	**.32**	**.58**
7. To what extent has the threat of coronavirus influenced your decisions to be around people	.19	**.68**
8. To what extent has the threat of coronavirus influenced your use of safety behaviors (e.g., hand sanitizer)	-.02	**.76**
9. To what extent has the threat of coronavirus influenced your travel plans	-.06	**.82**

Note. F1 = Perceived Vulnerability; F2 = Threat Response. Factor loadings greater than .30 are bold.

[a] Items 5 and 6 had double loadings.

Table S2. *Descriptive Statistics and Tucker's Phi for Perceived Vulnerability and Threat Response across Countries/Societies*

		Perceived vulnerability				Threat response			
	n	*α*	*M*	*SD*	*phi*	*α*	*M*	*SD*	*phi*
Argentina	522	.77	2.47	0.63	0.99	.68	3.39	0.74	0.97
Australia	515	.80	2.68	0.67	0.99	.68	3.26	0.76	0.98
Brazil	530	.76	2.99	0.62	0.98	.63	3.40	0.71	0.96
Canada	526	.80	2.79	0.65	0.98	.66	3.33	0.72	0.97
China	519	.81	2.24	0.68	1.00	.71	3.17	0.67	0.98
Egypt	516	.85	2.49	0.71	0.99	.74	3.30	0.71	0.99
Finland	518	.80	2.71	0.60	1.00	.73	3.30	0.74	0.99
France	523	.73	3.02	0.53	0.96	.70	3.29	0.77	0.93
Germany	528	.73	2.69	0.59	0.90	.64	3.06	0.81	0.78
Hong Kong	526	.77	2.49	0.60	1.00	.73	3.29	0.62	0.99
India	519	.81	2.70	0.73	0.98	.69	3.30	0.66	0.96
Indonesia	526	.79	2.95	0.63	0.99	.75	3.49	0.56	0.98
Italy	527	.73	3.04	0.54	0.99	.62	3.41	0.64	0.98
Japan	515	.77	3.04	0.55	0.98	.72	3.19	0.74	0.99
Malaysia	525	.79	2.73	0.65	0.99	.69	3.48	0.59	0.99
Mexico	525	.80	2.71	0.67	1.00	.69	3.40	0.70	0.99
Netherlands	511	.72	2.90	0.52	0.98	.66	3.05	0.76	0.98
New Zealand	507	.78	2.45	0.66	1.00	.63	3.15	0.78	1.00
Nigeria	516	.80	2.17	0.69	1.00	.73	3.38	0.72	1.00
Pakistan	518	.79	2.29	0.68	1.00	.70	3.04	0.74	1.00
Philippines	510	.77	2.76	0.67	0.99	.72	3.54	0.61	0.99
Portugal	511	.69	2.94	0.54	0.97	.53	3.52	0.58	0.98
Russia	517	.83	2.49	0.71	1.00	.73	3.05	0.83	0.99
South Africa	523	.75	2.80	0.65	1.00	.62	3.51	0.64	0.99
South Korea	523	.79	2.48	0.62	1.00	.77	3.23	0.69	0.99
Singapore	524	.80	2.78	0.59	1.00	.63	3.31	0.62	1.00
Spain	518	.74	3.30	0.50	1.00	.53	3.38	0.66	1.00
Sweden	510	.79	2.90	0.64	0.99	.69	3.09	0.80	0.99
Taiwan	515	.82	1.97	0.66	0.99	.73	3.13	0.74	0.97
Thailand	516	.74	2.73	0.60	0.97	.72	3.40	0.61	0.96
Turkey	515	.83	2.84	0.65	1.00	.58	3.48	0.57	1.00
UAE	525	.82	2.56	0.71	1.00	.74	3.45	0.63	1.00
UK	523	.80	3.11	0.59	0.99	.66	3.45	0.65	0.99
USA	516	.81	2.97	0.67	0.97	.71	3.28	0.77	0.94
Vietnam	513	.85	2.42	0.76	1.00	.71	3.31	0.63	1.00

Table S3. *Percentages of Normal, Mild, Moderate, Severe, and Extremely Severe Depression*

	Normal	Mild	Moderate	Severe	Extremely Severe
Argentina	63.9	13.0	12.3	5.0	5.8
Australia	62.5	10.4	13.6	4.6	8.9
Brazil	49.3	16.8	16.1	7.7	10.1
Canada	57.6	12.0	16.3	6.0	8.2
China	62.0	7.9	15.0	7.7	7.4
Egypt	46.8	16.6	21.5	7.4	7.8
Finland	59.0	11.5	17.0	5.4	7.1
France	64.6	12.0	13.6	4.6	5.2
Germany	67.3	10.8	12.9	4.5	4.6
Hong Kong	56.5	14.6	16.7	7.5	4.7
India	49.7	9.1	17.4	12.7	11.0
Indonesia	76.5	7.1	9.4	3.3	3.8
Italy	58.5	16.1	14.9	6.1	4.4
Japan	68.0	8.9	15.6	3.8	3.7
Malaysia	60.8	13.1	14.4	5.5	6.3
Mexico	73.7	9.5	9.7	3.4	3.7
Netherlands	72.9	10.2	9.7	4.3	2.9
New Zealand	62.9	15.8	12.8	4.7	3.9
Nigeria	76.5	7.8	10.5	2.6	2.6
Pakistan	59.5	12.4	12.8	9.8	5.6
Philippines	60.3	11.4	17.1	6.3	4.9
Portugal	56.3	17.9	15.3	6.9	3.6
Russia	54.9	14.8	16.5	8.6	5.3
South Africa	58.1	10.8	16.0	7.7	7.5
South Korea	53.1	14.2	20.5	6.4	5.8
Singapore	59.3	13.4	13.4	6.3	7.6
Spain	72.6	9.2	9.9	3.8	4.5
Sweden	56.9	10.9	16.3	6.9	9.0
Taiwan	59.1	16.0	15.5	5.2	4.1
Thailand	58.5	16.2	15.5	4.8	5.0
Turkey	51.1	13.8	19.0	8.7	7.4
UAE	59.8	9.3	17.9	5.2	7.8
UK	59.6	13.8	13.1	5.8	7.7
USA	61.7	9.3	12.5	7.9	8.6
Vietnam	68.0	13.6	9.8	3.8	4.9

Table S4. *Percentages of Normal, Mild, Moderate, Severe, and Extremely Severe Anxiety*

	Normal	Mild	Moderate	Severe	Extremely Severe
Argentina	72.1	6.1	12.0	3.0	6.8
Australia	72.5	4.8	8.2	4.3	10.3
Brazil	59.2	8.2	13.1	5.9	13.6
Canada	67.2	4.6	12.0	5.4	10.8
China	57.2	8.1	14.1	5.7	14.9
Egypt	45.2	10.8	19.2	9.8	15.0
Finland	68.2	7.8	13.6	5.4	5.1
France	76.8	5.8	9.9	3.1	4.4
Germany	74.3	5.7	8.8	4.5	6.8
Hong Kong	60.1	7.5	16.1	7.3	8.9
India	41.8	8.9	17.4	7.2	24.8
Indonesia	56.4	11.0	18.9	4.9	8.8
Italy	77.4	5.7	8.1	2.4	6.4
Japan	82.0	4.0	8.5	2.9	2.7
Malaysia	57.9	8.5	16.6	7.2	9.8
Mexico	72.5	6.6	10.9	4.3	5.8
Netherlands	77.8	5.4	10.4	1.6	4.7
New Zealand	74.9	6.7	10.0	3.5	4.9
Nigeria	68.8	8.0	12.1	4.8	6.4
Pakistan	54.2	5.8	17.4	6.3	16.3
Philippines	51.0	8.7	20.1	6.5	13.7
Portugal	70.2	8.2	12.0	3.8	5.9
Russia	64.1	8.1	15.5	4.3	8.0
South Africa	62.9	7.3	14.3	4.4	11.1
South Korea	62.5	8.9	17.4	4.7	6.6
Singapore	62.5	8.4	17.1	3.9	8.1
Spain	74.7	6.0	11.6	3.7	4.1
Sweden	67.7	6.1	11.9	5.5	8.8
Taiwan	61.2	9.4	15.9	5.8	7.7
Thailand	47.2	11.0	21.8	8.8	11.1
Turkey	63.7	7.5	16.2	4.5	8.2
UAE	51.5	12.7	17.7	5.8	12.3
UK	71.0	6.4	10.9	4.8	7.0
USA	64.5	7.1	10.9	4.5	13.1
Vietnam	54.3	8.8	21.2	8.1	7.6

Table S5. *Percentages of Normal, Mild, Moderate, Severe, and Extremely Severe Stress*

	Normal	Mild	Moderate	Severe	Extremely Severe
Argentina	73.9	10.5	7.1	5.6	3.0
Australia	74.5	6.7	9.2	7.1	2.6
Brazil	59.0	11.2	11.5	11.2	7.1
Canada	69.7	8.7	11.5	7.7	2.5
China	70.8	8.1	9.2	10.1	1.8
Egypt	51.4	16.3	15.3	12.1	4.9
Finland	75.2	9.3	8.2	4.9	2.5
France	79.3	7.7	6.1	4.8	2.1
Germany	75.0	9.5	9.4	3.9	2.3
Hong Kong	70.5	10.0	12.1	6.4	1.0
India	60.9	9.4	10.7	15.3	3.8
Indonesia	70.9	10.4	11.0	6.4	1.3
Italy	74.1	9.9	9.9	4.4	1.8
Japan	78.4	8.5	8.2	4.0	0.9
Malaysia	72.5	11.7	7.7	5.5	2.6
Mexico	81.1	7.7	5.9	2.6	2.7
Netherlands	82.4	6.1	7.0	3.3	1.2
New Zealand	81.4	7.9	5.0	3.6	2.2
Nigeria	83.1	7.5	5.4	3.2	0.8
Pakistan	70.6	11.2	8.8	7.6	1.9
Philippines	73.3	9.7	11.2	4.6	1.2
Portugal	64.1	14.5	13.2	6.0	2.2
Russia	64.0	10.7	11.7	8.4	5.2
South Africa	69.9	10.5	11.0	6.3	2.4
South Korea	68.1	13.8	11.3	6.0	0.9
Singapore	74.4	8.8	9.6	6.0	1.3
Spain	79.3	7.3	7.5	4.7	1.2
Sweden	71.1	9.2	10.3	5.7	3.8
Taiwan	72.5	12.8	10.0	3.2	1.6
Thailand	64.6	14.6	11.7	6.4	2.7
Turkey	68.3	11.4	11.0	5.8	3.5
UAE	67.1	11.7	12.2	6.4	2.7
UK	76.9	9.2	6.5	4.9	2.4
USA	70.9	7.4	10.8	7.3	3.6
Vietnam	71.8	10.1	10.6	4.9	2.6

Table S6. *Multilevel Regression Predicting Perceived Vulnerability and Threat Response by Need for Cognitive Closure at the Society Level*

	Perceived vulnerability	*Threat response*
	β	β
1. Age	-.16	-.77***
2. Gender	.06	.02
3. Education level	-.65***	-.62***
4. Need for cognitive closure	.01	.19

***$p < .001$

Table S7. *Multilevel Regression Predicting Well-being Outcomes by Perceived Vulnerability and Threat Response at the Society Level*

	Self-rated health	*Subjective well-being*	*Negative emotional symptoms*	*Psychological distress*
	β	β	β	β
1. Age	-.83**	-.52*	-.49	-.27
2. Gender	-.07	-.13	.06	-.04
3. Education level	-.19	.18	.23	.28
4. Perceived vulnerability	.12	.02	.04	.25
5. Threat response	.03	.10	-.07	.15

*$p < .05$, **$p < .01$.

Table S8. *Multilevel Regression Predicting Well-being Outcomes by Perceived Vulnerability and Threat Response at the Individual Level (Controlling for Extreme Response Style)*

	Self-rated health	Subjective well-being	Negative emotional symptoms	Psychological distress
	β	β	β	β
Age	-.11***	.11***	-.20***	-.22***
Gender [a]	-.03**	-.03**	.04**	.07***
Education level	.09***	.08***	-.05***	-.05***
Perceived vulnerability	-.17***	-.10***	.23***	.21***
Threat response	.07***	.12***	-.08***	-.06***
Extreme response style [b]	.09***	.12***	.02	.01

Note. [a] Male = reference group. [b] Extreme response style is tapped by the percentage of 1s and 7s across six unrelated 7-point scale items in the survey.

**p* < .05, ***p* < .01, ****p* < .001.

Table S9. *Multilevel Regression Predicting Perceived Vulnerability and Threat Response by Need for Cognitive Closure at the Individual Level (Controlling for Extreme Response Style)*

	Perceived vulnerability	Threat response
	β	β
Age	-.02	.04**
Gender [a]	.07***	.09***
Education level	.04***	.08***
Need for cognitive closure	.10***	.09***
Extreme response style [b]	.08***	.06***

Note. [a] Male = reference group. [b] Extreme response style is tapped by the percentage of 1s and 7s across six unrelated 7-point scale items in the survey.

***p* < .01, ****p* < .001.
